# A retrospective European multicenter analysis of the functional outcomes after active middle ear implant surgery using the third generation vibroplasty couplers

**DOI:** 10.1007/s00405-020-06064-x

**Published:** 2020-05-25

**Authors:** Torsten Rahne, Piotr Henryk Skarzynski, Rudolf Hagen, Andreas Radeloff, Luis Lassaletta, Maurizio Barbara, Stefan K. Plontke, Robert Mlynski

**Affiliations:** 1 Department of Otorhinolaryngology, Head and Neck Surgery, Martin Luther University Halle-Wittenberg, University Medicine Halle (Saale), Halle (Saale), Germany; 2 Department of Teleaudiology and Screening, World Hearing Center, Institute of Physiology and Pathology of Hearing, Warsaw, Poland; 3 Heart Failure and Cardiac Rehabilitation Department, 2nd Faculty of Medicine, Medical University of Warsaw, Warsaw, Poland; 4 Institute of Sensory Organs, Kajetany, Poland; 5 Department of Otorhinolaryngology, Head and Neck Surgery, University Hospital Würzburg, Würzburg, Germany; 6 Department of Otorhinolaryngology, University of Oldenburg, Oldenburg, Germany; 7 Department of Otolaryngology, La Paz University Hospital, Centro de Investigación Biomédica en Red de Enfermedades Raras (CIBERER-U761), Instituto de Investigación Hospital Universitario La Paz (IdiPAZ), Madrid, Spain; 8 NESMOS Department, Otorhinolaryngology Clinic, University Hospital Sant’Andrea, Sapienza University, Rome, Italy; 9 Department of Otorhinolaryngology, Head and Neck Surgery “Otto Körner”, University Medical Center Rostock, Rostock, Germany

**Keywords:** Vibroplasty, Couplers, Active middle ear implant, Vibrant Soundbridge, Hearing loss

## Abstract

**Purpose:**

To evaluate the safety and performance of three novel vibroplasty couplers that allow attachment of the floating mass transducer of a transcutaneous active middle ear implant (AMEI) to the round window (RW) membrane, the long process (LP), or the incus body and the short process (SP) of the incus.

**Methods:**

Retrospective multicenter cohort study of 25 AMEI users with sensorineural or mixed hearing loss that were among the first implanted with an AMEI vibrating ossicular prosthesis in combination with the third generation of vibroplasty couplers between 2014 and 2016. Main Outcome Measures were bone-conduction pure-tone and vibroplasty thresholds, postoperative aided sound field thresholds and postoperative aided word recognition score (WRS).

**Results:**

Bone conduction threshold changes of more than 10 dB in 4PTA_BC_ were observed in two subjects. A mean improvement of 57.8% in speech recognition was observed with a mean WRS at 65 dB SPL improving from 14.8% (SD 21.9%) preoperatively to a mean aided score of 72.6% (SD 18.6%). Sound field thresholds improved from an average 4PTA_SF_ of 64.1 dB HL (SD 9.8 dB HL) to 37.0 dB HL (SD 8.9 dB HL), resulting in a mean functional gain of 27.1 dB. There was no significant difference in WRS or functional gain between the coupler types.

**Conclusion:**

Initial experience shows that all three third generation vibroplasty couplers represent safe and efficient attachment options for the FMT allowing the surgeon to choose the coupling type based on the present pathology.

## Introduction

The transcutaneous, semi-implantable active middle ear implant (AMEI) provides an alternative treatment option for patients with sensorineural (SNHL), conductive (CHL) and mixed hearing losses (MHL) who cannot wear conventional acoustic hearing aids (HA) for medical reasons, or who are unsuccessful acoustic HA users, or who do not experience adequate benefit from their device [1]. The AMEI includes an external part, the audio processor (AP), and an implanted part, the vibrating ossicular prosthesis (VORP) consisting of a receiver/stimulator, a conductor link, and a floating mass transducer (FMT). Information from the AP is sent to the VORP so that the FMT vibrates a mobile structure of the middle ear (i.e. the incus, the stapes suprastructure, or the stapes footplate) or the inner ear (i.e. the round window membrane) and thus stimulates the cochlear fluids.

The surgical treatment of hearing loss via vibratory stimulation in the middle ear by using an active middle ear implant is termed vibroplasty [2, 3]. The classical approach with the FMT attached to the long process of the incus was first introduced in 1996 to treat patients with moderate to-severe SNHL [4, 5]. Additional coupling techniques were developed leading to new applications of the AMEI also allowing the treatment of CHL and MHL. Techniques for various FMT placements with and without couplers have been summarized in detail and proven to be stable over time [6–8].

**Fig. 1:**
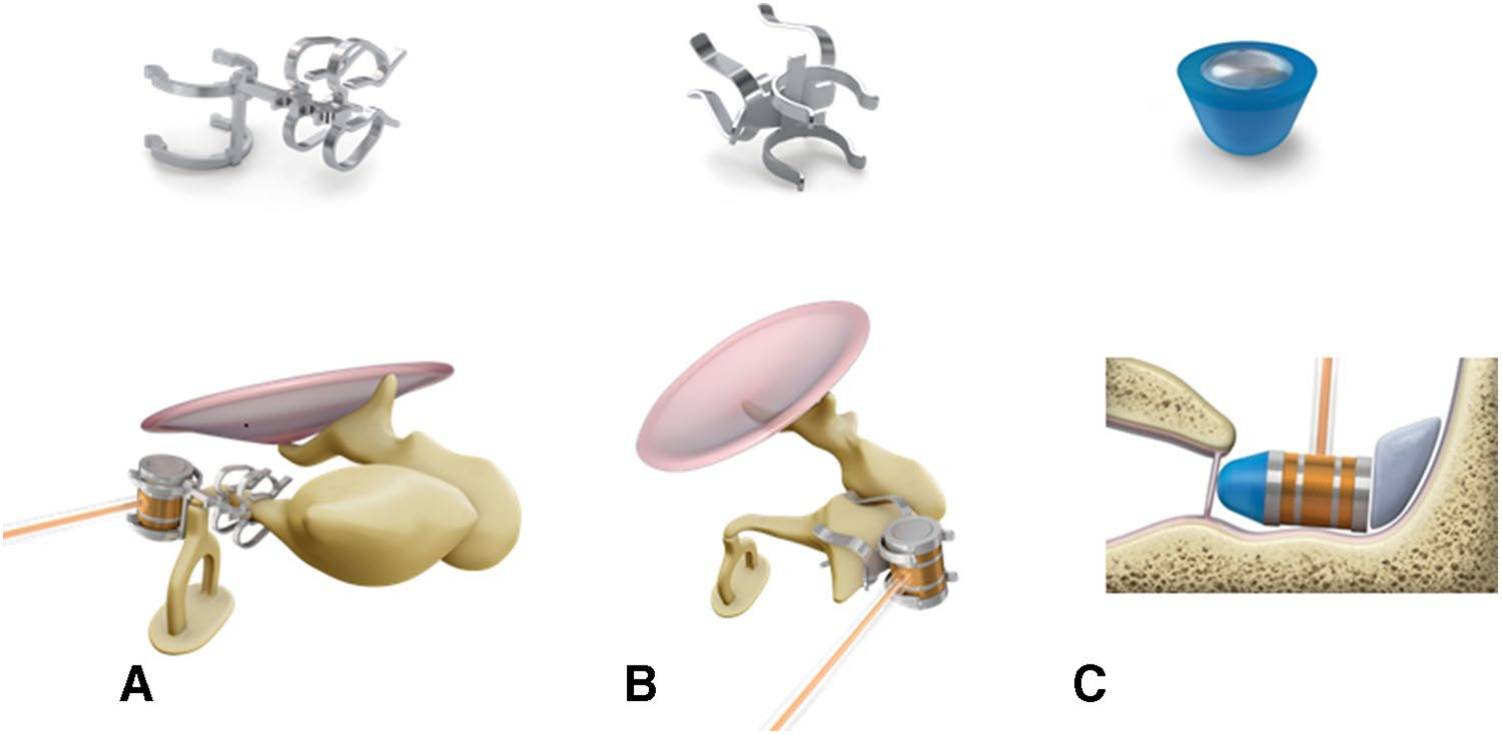
Coupler types and placement. **a** LP-coupler, **b** SP-coupler, **c** RW-soft-coupler

The present study assessed safety and performance of the third generation of vibroplasty couplers—the incus long process (LP)-coupler (Fig. 1a), the incus short process (SP)-coupler (Fig. 1b) and the round window-soft (RWS)-coupler (Fig. 1c)—in combination with the AMEI based on the functional outcomes of the first implantees in the participating study centers. The LP-coupler is attached to the long process of the incus and features a new attachment clip design that does not require crimping but is clipped onto the long process of the incus [9, 10]. Coupling of the FMT to the incus body and the short process of the incus represents an alternative to the classical coupling to the long process [11]. SP vibroplasty is performed without a posterior tympanotomy, but only requires a mastoidectomy with wide epitympanotomy for FMT attachment reducing the potential risk for facial nerve injury and reducing surgical time [12]. The first round window (RW) vibroplasty with the FMT in the RW niche was performed in 2005 [13]. That stimulation used fascia between the FMT and the RW membrane. Direct RW stimulation without fascia or other materials interposed between FMT and RW membrane also gave stable hearing results [14, 15]. Later, a titanium RW coupler was introduced to provide a better connection by adapting the geometries of the FMT to the smaller RW and reducing the drilling effort at the RW niche [16, 17]. The improved RWS-coupler is made out of silicone instead of titanium and provides an attachment of the FMT via a sticky pad.

Data on the performance of the SP-coupler [12, 18–22] and RWS-coupler [23] are limited and from single centers only. To our knowledge, no information on the clinical performance of the LP-coupler has been published so far. The aim of this study was to compare these three vibroplasty couplers and to investigate, if the location of the FMT has an impact on the audiological outcomes in a multicenter design.

## Materials and methods

### Study subjects

This was a retrospective analysis of patients implanted with the Vibrant Soundbridge at five centers in four European countries between 2014 and 2016: Halle/Saale (Germany), Würzburg (Germany), Warsaw/Kajetany (Poland), Madrid (Spain), and Rome (Italy). It was designed and executed in accordance with the principles of the Declaration of Helsinki. For the retrospective analysis no specific vote from the ethic committees was required. Only users of the Vibrant Soundbridge with the Amadé audio processor that were implanted with the VORP 502 (MED-EL, Innsbruck, Austria) in combination with the LP-coupler, SP-coupler or RWS-coupler were included in the study. Subjects were included irrespectively of the specific Amadé audio processor variant used. Due to the retrospective design of the study, audiological or etiologic data are not complete for all study patients. An incomplete data set was not a reason for exclusion of the patient, the minimum data requirement was the availability of aided sound field thresholds at least one post-operative measurement.

### Audiometric testing

At all study sites all audiometric data were recorded in sound-attenuated chambers according to the ISO requirements. Routine pure-tone audiometry was performed preoperatively for baseline measurement and 6–12 months after surgery. Both ears of each patient were evaluated using standard air conduction (AC) and bone conduction (BC) pure tone audiometry. Third-octave band noise was used for masking if applicable. The pure tone averages (4PTA_AC_ and 4PTA_BC_) were calculated across conversational frequencies 0.5, 1, 2, and 4 kHz. Based on the test–retest variability a reduction up to 10 dB in mean pure tone thresholds (4PTA) between pre- and 12-months post-operative outcomes will not be considered as clinically significant [24].

Postoperative assessments: Following clinical routine measurements, at the implanted side, sound field (SF) thresholds were measured using third-octave band noise with the center frequencies of 0.25, 0.5, 1, 2, 3, 4, 6 and 8 kHz. The contralateral side was plugged and covered. SF thresholds were determined preoperatively, at the fitting of the audio processor, i.e. 6–8 weeks after surgery and postoperatively at 6–12 months. The pure tone average (4PTA_SF_) was calculated across conversational frequencies 0.5, 1, 2, and 4 kHz. The functional gain (FG) was defined as the mean difference between unaided and aided 4PTA_SF_ [25]. The effective gain was determined by subtracting the aided 4PTA_SF_ and the pre-operative 4PTA_BC_ [26].

Sound field speech intelligibility was determined at sound pressure levels (SPL) of 65 and 80 dB using the monosyllable word recognition test subject to the native language of the patient. The languages included Polish, German, Spanish and Italian. Word recognition scores (WRS) with the contralateral side plugged and covered were determined preoperatively, at fitting and 6–12 months after surgery.

Vibrogram thresholds were obtained through direct stimulation of the implant as an in situ measurement as described previously [10, 27]. The vibrogram pure tone thresholds were determined at all frequencies at fitting and 6–12 months postoperatively. The pure tone average (4PTA_V_) was calculated across conversational frequencies 0.5, 1, 2, and 4 kHz.

### Statistical analyses

Non-parametric Wilcoxon signed-rank test was used to test for significant differences between two test conditions in the audiological measurements. Kruskal–Wallis test was used to assess the statistical significance between coupling methods. Statistical significance was defined as *p* < 0.05. GraphPad Prism 6 for Windows 2013, Version 6.02, was used for the analyses as well as the graphs.

## Results

### Patients

Twenty-five patients (9 male, 16 female) with MHL or SNHL were included in the study (see Table 1). The mean age of the patients was 51.7 years [standard deviation (SD) 16.9 years, range 10–72 years]. All subjects were implanted with the AMEI in combination with the SP-coupler, the LP-coupler or the RWS-coupler between September 2014 and September 2016. The respective surgeon chose the type of coupler based on the medical or otological preoperative and intraoperative findings. Four subjects were implanted with the AMEI using the LP-coupler, 12 with the SP-coupler and nine with the RWS-coupler.

**Table 1:** Patients’ characteristics

Patient	Language	Side implanted	Age (years)	Type of HL	Etiology of HL	4PTA_AC_ (dB HL)	4PTA_BC_ (dB HL)	Coupler type	4PTA_SF_ (dB HL)	WRS at 65 dB SPL (%)
ID01	German	R	54	SNHL	N/A	63.8	56.7	Incus-LP-Coupler	67.5	15
ID02	German	R	55	MHL	N/A	63.8	45.0	RW-Soft-Coupler	68.8	0
ID03	German	L	47	MHL	Otosclerosis	38.8	23.8	Incus-SP-Coupler	41.3	55
ID04	German	R	56	MHL	Otosclerosis	63.8	28.8	RW-Soft-Coupler	65.0	0
ID05	German	L	59	SNHL	SNHL and EAC stenosis	42.5	37.5	Incus-LP-Coupler	56.3	60
ID06	German	L	43	MHL	Previous tympanoplasty	71.3	33.8	RW-Soft-Coupler	N/A	0
ID07	German	R	65	MHL	Multiple surgeries	48.8	25.0	RW-Soft-Coupler	N/A	N/A
ID08	German	R	65	MHL	N/A	75.0	36.3	RW-Soft-Coupler	N/A	0
ID09	German	L	50	MHL	Cholesteatoma	60.0	17.5	RW-Soft-Coupler	41.3	0
ID10	Italian	R	64	SNHL	N/A	42.5	37.5	Incus-SP-Coupler	53.8	40
ID11	Polish	L	65	MHL	Chronic otitis media, Cholesteatoma	71.3	33.8	RW-Soft-Coupler	72.5	0
ID12	Polish	L	44	MHL	Chronic otitis media	66.7	18.3	RW-Soft-Coupler	N/A	N/A
ID13	Polish	L	28	MHL	Acquired atresia of EAC	62.5	27.5	Incus-SP-Coupler	57.5	0
ID14	Polish	R	57	SNHL	N/A	50.0	45.0	Incus-SP-Coupler	N/A	0
ID15	Polish	R	61	MHL	N/A	62.5	16.3	RW-Soft-Coupler	N/A	0
ID16	Polish	R	66	SNHL	N/A	43.8	40.0	Incus-LP-Coupler	52.5	55
ID17	Polish	R	57	SNHL	N/A	67.5	62.5	Incus-SP-Coupler	N/A	0
ID18	Polish	R	63	SNHL	N/A	52.5	51.3	Incus-SP-Coupler	62.5	25
ID19	Polish	L	63	SNHL	N/A	35.0	30.0	Incus-SP-Coupler	56.3	40
ID20	Polish	L	62	SNHL	Chronic otitis media	46.3	41.3	Incus-SP-Coupler	56.3	65
ID21	Polish	L	10	MHL	Bilateral atresia of EAC	55.0	15.0	Incus-SP-Coupler	58.8	0
ID22	Polish	L	23	MHL	N/A	77.5	31.3	Incus-SP-Coupler	60.0	10
ID23	Polish	L	46	SNHL	N/A	62.5	51.3	Incus-SP-Coupler	42.5	25
ID24	Polish	L	72	MHL	N/A	58.8	32.5	Incus-LP-Coupler	70.0	N/A
ID25	Spanish	R	35	MHL	N/A	66.6	58.3	Incus-SP-Coupler	N/A	35

Average pre-operative air and bone conduction thresholds (4PTA_AC_, 4PTA_BC_), sound field thresholds (4PTA_SF_) and unaided word recognitions scores (WRS) of the subjects

*HL* hearing loss, *R* right, *L* left, *SNHL* sensorineural hearing loss, *MHL* mixed hearing loss, *CHL* conductive hearing loss, *EAC* external auditory canal, *N/A* no information available

### Outcome assessment

Subjects were followed up for up to 1 year post surgery. Analysis of ten subjects with 6 and 12 month data showed that the mean WRS at 6 months was [61.5% (SD 30.4%) at 65 dB SPL and 82.5% (SD 17.0%) at 80 dB SPL] was not significantly different from the mean WRS at 12 month [68.5% (SD 29.5%) at 65 dB SPL and 79.0% (SD 24.2%) at 80 dB SPL], respectively (*p* = 0.24 for 65 dB SPL and *p* = 0.44 for 80 dB SPL). Thus, if measurements were available at 6 and 12 months postoperatively the latest available data were used for analysis of the postoperative performance.

### Safety

**Fig. 2:**
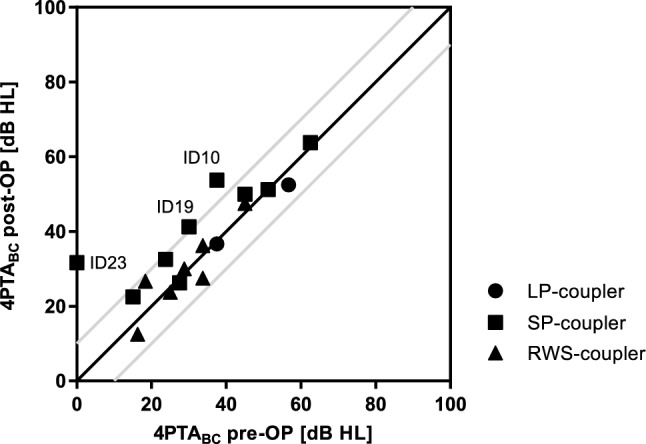
Changes in bone conduction thresholds. Preoperative and the latest available postoperative measurement for 4PTA_BC_. Changes above the 10 dB test–retest range (gray lines) are considered significant

Information on postoperative bone conduction was available for 17 of 25 subjects. The mean preoperative 4PTA_BC_ was 34.6 dB HL (SD 13.9) and was not significantly different from the postoperative 4PTA_BC_ of 37.3 dB HL (SD 13.9) (*p* = 0.57). However, in two subjects implanted with a SP-coupler the 4PTA_BC_ threshold deteriorated more than 10 dB (Fig. 2). No device or surgery related reasons were reported. There was no significant difference between the three couplers regarding preservation of residual hearing (mean 4PTA_BC_, *p* = 0.24).

### Speech recognition

**Fig. 3:**
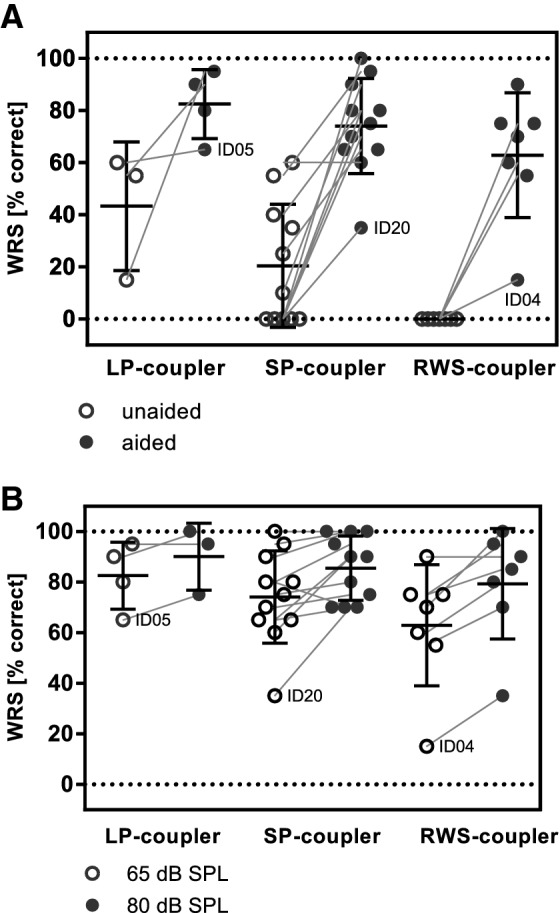
Speech recognition. **a** Unaided (latest available measurement, open symbols) and AMEI aided (postoperative, closed symbols) WRS at 65 SPL. **b**. Postoperative AMEI aided WRS at 65 (open symbols) and 80 dB SPL (closed symbols). Horizontal dotted lines indicate the mean WRS and standard deviation. No significant difference in WRS was observed between coupler types at 65 dB SPL (Kruskal–Wallis test, *p* = 0.23). Please note that the figure assembles results of speech recognitions tests from different languages

Aided word recognition scores measured at least 6 months after surgery were available for 21 subjects. Starting from a mean WRS of 14.8% (SD 21.9%) preoperatively speech understanding improved to a mean aided WRS of 72.6% (SD 18.6%) at 65 dB SPL. At 80 dB SPL a mean aided WRS of 83.8% (SD 16.4%) was reached. In the LP-coupler group the mean WRS improved from 43.3% (SD 24.7%) to 82.5% (SD 13.2%), in the SP-coupler group from 16.5% (SD 20.7%) to 75.5% (SD 20.7%) and in the RWS-coupler group from 0.0% (SD 0.0%) to 62.9% (SD 24.0%) at 65 dB SPL (Fig. 3a). An aided WRS at 65 dB SPL of at least 75% was reached by 3 of 4 subjects in the LP coupler group, 6 of 10 subjects in the SP-coupler group and 3 of 7 subjects in the RWS-coupler group. There was no significant difference in the post-operative aided WRS between the three coupler types at 65 dB SPL (*p* = 0.23) or at 80 dB SPL (*p* = 0.91) (Fig. 3b). The mean improvement in WRS at 65 dB SPL was 39.2% (percentage points) in the LP-coupler group, 59.0% in the SP-coupler group and 62.9% in the RWS-coupler group. An improvement of at least 20% was reached by 3 of 4 subjects in the LP-coupler group, by 9 of 9 subjects in the SP-coupler group and by 4 of 5 subjects in the RWS group. Subjects with an improvement of less than 20% also (ID04, ID05, ID20) had the largest gap between 4PTA_V_ and 4PTA_BC_ in the respective coupler group (Fig. 5).

### Pure-tone audiometry

**Fig. 4:**
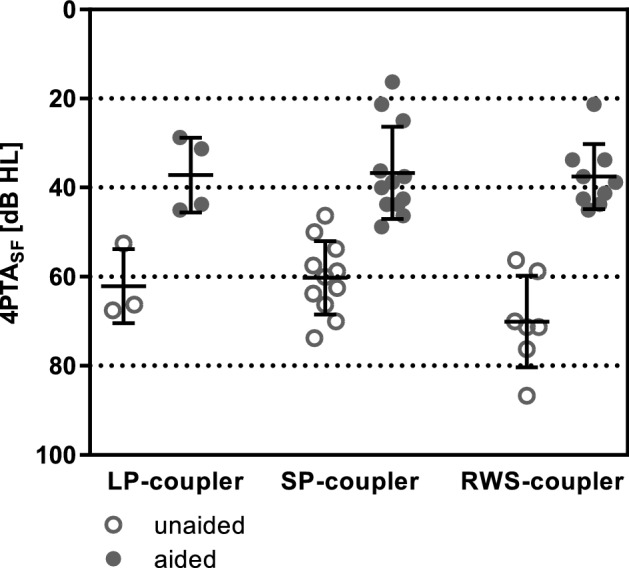
Sound field thresholds. Preoperative unaided 4PTA_SF_ (open symbols) were compared to postoperative aided 4PTA_SF_ (closed symbols). Horizontal lines indicate the mean 4PTA_SF_ and standard deviation

Aided SF thresholds measured at least 6 months after surgery were available for 24 subjects. SF thresholds improved from an average 4PTA_SF_ of 64.1 dB HL (SD 9.8 dB HL) to 37.0 dB HL (SD 8.9 dB HL), resulting in a mean functional gain of 27.1 dB. There was no significant difference in the post-operative aided 4PTA_SF_ between coupler types (Kruskal–Wallis test, *p* = 0.97): LP-coupler 37.2 dB HL (SD 8.4 dB HL), SP-coupler 36.6 dB HL (SD 10.9 dB HL) and RWS-coupler 37.5 dB HL (SD 7.3 dB HL) (Fig. 4). The mean functional gain per coupler type was 27.1 dB (SD 9.0 dB, *N* = 3) for the LP-coupler, 24.6 dB (SD 8.0 dB, *N* = 10) for the SP-coupler and 33.5 dB (SD 7.9 dB, *N* = 7) for the RWS-coupler. Compared to the pre-operative bone conduction thresholds the effective gain in SF was in average + 2 dB (SD 14.4 dB). There was no significant difference in effective gain between the coupler types (*p* = 0.14): LP-coupler − 3.8 dB (SD 16.6 dB, *N* = 4), SP-coupler − 3.1 dB (SD 10.5 dB, *N* = 11) and RWS-coupler + 6.9 dB (SD 11.8 dB, *N* = 9).

**Fig. 5:**
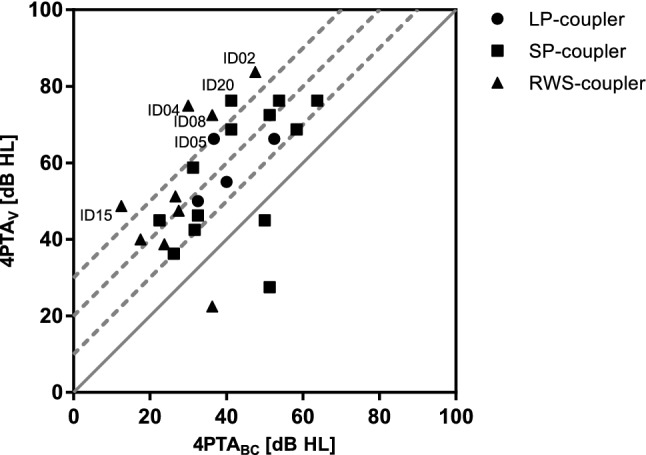
Coupling quality. The comparison of bone conduction and vibrogram thresholds can be used to assess coupling efficiency. An optimal coupling is indicated by the full line. The larger the perpendicular distance from this line to the left, the poorer is the coupling quality. Limited available data suggest that a difference of greater than 20 dB is associated with a higher risk of unsatisfactory word recognition scores [23]. The 10, 20 and 30 dB differences are shown in gray and dashed as guiding lines

The efficacy of vibroplasty (coupling efficiency) was assessed by comparing the 4PTA_V_ and 4PTA_BC_ from the latest available measurement (Fig. 5): higher thresholds in 4PTA_V_ compared to the 4PTA_BC_ indicate that coupling is suboptimal. For incus coupling (SP and LP couplers, *N* = 16) the offset was less than 20 dB in 9 subjects and 20–30 dB in 5 subjects and 30 dB or greater in 2 subjects. The two subjects (ID05 with LP-coupler and ID20 with SP-coupler) with a large gap of more than 30 dB showed lower performance and benefit in speech recognition compared to the other subjects. In RW coupling (*N* = 9 subjects) the difference between vibrogram and bone conduction thresholds was overall larger compared to incus coupling: the gap was less than 20 dB in 2 subjects and 20–30 dB in 3 subjects and 30 dB or greater in 4 subjects. Also, for coupling of the AMEI to the round window, the subject with the largest 4PTA gap of 45 dB (ID04) had the lowest performance in speech recognition and did not reach an improvement of 20%. The other three subjects (ID02, ID08, ID15), with a gap between 30 and 40 dB, achieved a WRS at 65 dB SPL of 55%, 75% and 75%, respectively.

## Discussion

This is the first report comparing the clinical performance of the LP-coupler, SP-coupler and RWS-coupler within one study. For the LP-coupler, up to now only data from temporal bone studies have been published [9, 10]. Within this study, four subjects were implanted with the novel LP-coupler. This coupler was developed to omit the crimping step required for classical coupling of the AMEI to the long process of the incus. Three of the four subjects (ID01, ID16, ID24) suffering from SNHL achieved an aided WRS of 80% and above with a mean WRS of 82.5% across all four subjects. Subject ID05 showed a gap above 30 dB between bone conduction and vibrogram thresholds, suggesting poor coupling of the FMT to the incus, and thus possibly explaining the lower benefit experienced by this patient. The functional gain in this group was 25 dB what is comparable to what was reported for a large study on audiological outcomes with the AMEI in SNHL [28] but has limited implication for patient satisfactory. For incus vibroplasty Maier and co-workers reported a short-term average functional gain of 21–24 dB and a mean WRS of 65–75% [28]. No change in bone conduction was observed.

Similar as for the LP-coupler, clinical data on the RWS-coupler are also limited. Only in one study [23] six subjects implanted with the AMEI in combination with the RWS-coupler were included. However, outcome data for WRS were not stratified according to coupler type. Nine patients with MHL were implanted with the RWS-coupler in combination with the AMEI in the current study with WRS data for 7 subjects at least 6 months after implantation. All but one subject (ID04) reached a post-operative WRS of more than 50%. Subject ID04 suffered from a MHL due to bilateral otosclerosis and achieved a WRS of 65% at 80 dB SPL at the initial activation. However, the WRS decreased over time and was only 35% at the 12-month evaluation. This subject may require a revision surgery as there was also a gap in vibroplasty and bone conduction thresholds of 65, 55, 35 and 25 dB at 0.5, 1, 2 and 4 kHz at the 12 months evaluation. After excluding subject ID04 from the analysis, the average WRS at 65 dB SPL in the RWS-coupler group was 71% (SD 12%) for six subjects. The improvement in WRS was 66% (SD 10%) compared to the pre-operative score considering only the four subjects where both data sets were available. This is comparable to the aided WRS of 73.3% reported for the titanium RW-coupler one year after surgery by Zahnert et al. [17]. Two subjects (ID08, ID09) scored 60 and 75% in WRS at 65 dB SPL at fitting, but were not evaluated at later time points and are thus not contained in the WRS analysis. The average functional gain in the RWS-coupler group was 36 dB (SD 10 dB) excluding subject ID04 from the analysis. This is slightly lower than the functional gain of 43 dB reported for the titanium RW-coupler [17], but comparable to the mean functional gain for RW coupling of 34 dB reported by Schraven et al. [7]. Regarding the quality of coupling, only 2 of 9 subjects of the RWS-coupler group had a difference of less than 20 dB between 4PTA_V_ and 4PTA_BC_ compared to 10 of 17 for incus coupling. Four of nine (33%) subjects (ID02, ID04, ID08, ID15) even showed a gap above 30 dB between postoperative vibrogram and bone conduction 4PTA. In general, no correlation was observed between postoperative WRS and the vibrogram gap, only the subject (ID04) with the largest difference had the lowest benefit. Overall, the offset between vibrogram and bone conduction thresholds, seems to be larger in round window vibroplasty, potentially due to lack of a rigid fixation mechanism to incus coupling. The association between RW coupling, a higher risk of poor coupling efficiency, and a deficit in reaching maximum word recognition score was previously observed [24]. However, no data on the preoperative maximum word recognition score were available in our patient group.

In contrast to the other two couplers the clinical performance of the SP-coupler has previously been reported. The main advantage of this coupler is, that for its placement only a simple mastoidectomy with a wide posterior epitympanotomy is required, substantially facilitating the surgical procedure for AMEI implantation. Lee et al. [21] reported that the surgical time could be reduced from 138 min (SD 34 min) for classical incus LP vibroplasty to 76 min (SD 25 min) for SP vibroplasty with the SP-coupler. To date, the scientific literature reports on a total of 26 patients who have received an AMEI in combination with a SP-coupler [11, 19–21, 23]. Out of twelve subjects that received the SP-coupler in the present study, seven suffered from SNHL. In this subgroup, a mean functional gain of 23 dB [from 66 dB (SD 5 dB) unaided to 43 dB (SD 5 dB) aided, *N* = 6] for 4PTA_SF_ and a mean WRS of 67% (SD 17%) at 65 dB SPL and 79% (SD 11%) at 80 dB SPL were measured. These results were comparable to published data: in patients with SNHL a functional gain of 14.9 dB (SD 5.6 dB) was observed and speech understanding improved from a WRS of 60.9% (SD 8.4%) at a most comfortable level (MCL) of 85.1 dB (SD 9.1 dB) to an aided WRS of 65.4% (SD 16.8%) at a MCL of 56.7 dB (SD 4.0 dB) [21]. Although the SP-coupler is indicated for SNHL also five subjects suffering from MHL were implanted with the AMEI via the SP-coupler in the present study. In patients with MHL a functional gain of 27 dB (*N* = 4) was achieved in the SP-coupler group: SF thresholds improved from 52 dB (SD 5 dB) to 29 dB (SD 12 dB). This is comparable to the SNHL group in this study, but was lower than previously reported: for patients with MHL and CHL implanted with a SP-coupler a functional gain of 42.2 dB (SD 7.3 dB) was described [20]. However, the study populations had a different preoperative air–bone gap with 29 dB (SD 16 dB) in our study and 51.3 dB (SD 8.9 dB) in the study by Thomas et al. [20]. The effective gain seemed to be slightly better with − 1.9 dB (SD 11.2 dB) in this study and + 9.0 dB (SD 13.4 dB) in the published data set. A mean WRS of 89% (SD 13%) was observed at 65 dB SPL and of 94% (SD 13%) at 80 dB SPL in the MHL subgroup of the SP-coupler group.

Nine of 21 subjects did not achieve a WRS at 65 dB SPL of 75% or higher: 1 of 4 subjects in the LP-coupler group, 4 of 10 subjects in the SP-coupler group and 4 of 7 subjects in the RWS-coupler group. However, there was also no information on the maximum pre-operative WRS available. An improvement of at least 20% was not reached in two patients: ID05 with an LP-coupler and ID04 with an RWS coupler. Thus, treatment success could not be correlated to the type of coupler selected by the surgeon. A previous study showed that the audiological outcome after vibroplasty depended on the coupling efficiency reflected by vibrogram thresholds. However, in this study, the offset between vibrogram and bone conduction thresholds was of limited benefit to predict successful hearing rehabilitation: only those three subjects with the largest gap of more than 30 dB in each coupler group also had the least benefit in speech understanding. Consequently, coupling of the AMEI to the respective mobile structure might not be firm enough in these cases for efficient transmission of vibrations from the FMT to the cochlea. These three patients potentially require repositioning of the FMT. However, no further correlation between vibrogram and bone conduction thresholds and speech understanding was observed in the absence of data on the preoperative maximum word recognition score. In case of adhesions around the FMT that limit the benefit from the device, adhesiolysis has been described as revision surgery techniques for coupling of the AMEI to the short process of the incus [29].

Changes in residual hearing could not be assessed for all patients as information on postoperative bone conduction thresholds was only available for 17 of 25 patients (68%) in this retrospective study. This should be more closely monitored in clinical practice. A recent publication highlights which audiological, surgical and subjective outcome measures should ideally be collected for the investigation of active middle ear implants [25]. This would facilitate the retrospective analysis of patient data and data pooling across studies. Bone conduction changes of more than 10 dB were observed in two subjects. Subject ID10 experienced a worsening of 16.3 dB 4PTA_BC_ and subject ID19 of 11.3 dB. These subjects still benefit from the AMEI with an aided WRS of 60–80% at 65 dB SPL. The change in BC thresholds could not be linked to the device or surgery, thus the cause remains unclear. Although both subjects were implanted with a SP-coupler, no significant difference in bone conduction changes was observed between the three coupling modalities.

Although this data set from 25 patients allows an initial assessment and comparison, we also have to acknowledge some limitations of the study: due to the retrospective nature of the study, not all data sets were complete. In addition, word recognition tests were done in different languages. Due to the differences in steepness of the discrimination curves and test–retest variability a direct comparison of the scores of different languages is not ideal. Based on these limiting factors, this study should be viewed as initial assessment.

In conclusion, the third generation LP and SP couplers provide a safe and efficient option for coupling the FMT of the AMEI to a vibratory structure of the middle ear. Whereas more experienced surgeons may prefer coupling to the long process of the incus, others may choose the SP-coupler combined with the benefit of a faster and easier surgery. Coupling to the RW may be necessary in challenging anatomical situations associated with malformations and disruptions of the ossicular chain and goes along with the risk for less effective coupling which reduces the audiological indication range. There was no significant difference in the mean audiological performance of the LP-coupler, SP-coupler or RWS-coupler in the groups of this initial study, suggesting that the surgeon may choose the most suitable coupler based on the specific medical and surgical condition of the patient’s ear.
